# Comparison of Tibial Cortical Button Fixation and Bioabsorbable Interference Screw Plus U-Staple Fixation in Anterior Cruciate Ligament Reconstruction: A Retrospective Cohort Study

**DOI:** 10.3390/medicina62091629

**Published:** 2026-08-25

**Authors:** Oktay Polat, Berk Koncalıoğlu, Mehmet Kuyumcu, Emrecan Akgün, Mohammed N. M. Ziara, Dursun Artvin, Sadret Mert Çınar, Osman Mert Topkar

**Affiliations:** 1 Orthopedics and Traumatology, Sultanbeyli State Hospital, Istanbul 34935, Turkey; dr.opolat89@gmail.com (O.P.);; 2 Orthopedics and Traumatology, Marmara University, Istanbul 34899, Turkey

**Keywords:** anterior cruciate ligament reconstruction, cortical button, U-staple, tibial fixation, hamstring autograft, graft diameter

## Abstract

*Background and Objectives:* This study aimed to compare the clinical, functional, and graft-related outcomes of two tibial fixation constructs used in primary hamstring autograft anterior cruciate ligament reconstruction (ACLR): tibial cortical button fixation and bioabsorbable interference screw fixation reinforced with a U-shaped staple. *Materials and Methods*: This retrospective two-center comparative cohort study included 58 patients with isolated anterior cruciate ligament injuries who underwent primary ACLR and had a minimum postoperative follow-up of 12 months. Patients were classified into the cortical button group (*n* = 26) or the U-staple group (*n* = 32) according to the initial graft diameter, which strictly dictated the tibial fixation construct documented in the operative report. Both the semitendinosus and gracilis tendons were used in each group. A shorter quadrupled graft construct was prepared in the cortical button group, whereas a longer doubled graft construct was used in the U-staple group. Donor-site pain measured using the visual analog scale (VAS), graft diameter, International Knee Documentation Committee (IKDC) score, Lysholm score, knee range of motion (ROM), Lachman and pivot-shift findings, and graft failure were compared between the groups. Graft failure was defined as magnetic resonance imaging (MRI) evidence of graft discontinuity or insufficiency accompanied by grade 2–3 Lachman laxity or a positive pivot-shift test. Numerical variables were analyzed using the Mann–Whitney U test, whereas categorical variables were compared using the Pearson chi-square or Fisher’s exact test, as appropriate. *Results*: Donor-site pain was significantly higher in the cortical button group than in the U-staple group [median VAS score: 2.5 (2.0–4.0) vs. 2.0 (0–2.0), respectively; *p* = 0.007]. Graft diameter was also significantly greater in the cortical button group [10.0 mm (10.0–10.0) vs. 9.0 mm (8.0–9.0); *p* < 0.001]. In contrast, IKDC scores were significantly higher in the U-staple group [71.0 (51.5–78.0) vs. 60.0 (55.0–62.8); *p* = 0.017]. No significant differences were observed in Lysholm scores (*p* = 0.701), knee ROM (*p* = 0.508), positive pivot-shift findings (11.5% vs. 6.3%; *p* = 0.648), clinically relevant Lachman laxity (30.8% vs. 18.8%; *p* = 0.287), or graft failure rates (11.5% vs. 18.8%; *p* = 0.495). *Conclusions*: No significant between-group differences were observed in Lysholm scores, knee ROM, clinical stability, or graft failure rates after hamstring autograft ACLR. The U-staple group demonstrated lower donor-site pain and higher IKDC scores, whereas the cortical button group had larger graft diameters, likely reflecting the shorter quadrupled graft configuration. Overall, neither tibial fixation construct showed a consistent advantage across all clinical outcomes.

## 1. Introduction

Anterior cruciate ligament (ACL) reconstruction (ACLR) is widely performed in active patients with symptomatic instability to restore functional knee stability. In hamstring autograft procedures, fixation strength is especially important during the early postoperative period, before biological graft incorporation within the bone tunnels has occurred. The tibial side represents a particular concern because metaphyseal bone density is relatively low and soft-tissue grafts lack a bone block, increasing the potential for graft slippage and construct elongation. Previous biomechanical studies have reported marked differences among tibial fixation devices in terms of stiffness, cyclic displacement, and ultimate failure load, highlighting the importance of achieving reliable tibial fixation in ACLR [1,2].

Bioabsorbable interference screws provide aperture fixation by compressing the graft against the tibial tunnel wall and are commonly used in hamstring autograft ACLR. However, their fixation performance may be affected by tibial bone quality, insertion technique, and mismatch between graft and tunnel diameters. For this reason, additional extracortical fixation is often used to reinforce the tibial construct. U-shaped staples are inexpensive and technically straightforward, and some studies have reported improved anterior stability when they are combined with an interference screw. Nevertheless, the clinical benefit of routine staple augmentation remains uncertain, particularly because implant prominence, local tenderness, and discomfort during kneeling may occur [3,4,5]. Recent pooled evidence suggests that supplementary tibial fixation may offer only a modest improvement in stability while increasing the risk of postoperative implant-related pain [6].

Cortical button fixation provides an alternative tibial fixation strategy by transferring the graft load to the cortical surface. In short-graft or all-inside-type constructs, cortical suspensory fixation permits the use of a shorter graft, allowing the combined hamstring graft to be prepared in a shorter quadrupled configuration and potentially resulting in a larger graft diameter. By contrast, the conventional full-tunnel construct used with a bioabsorbable interference screw and U-shaped staple requires sufficient graft length for tunnel passage and fixation [7]. Although both techniques can provide satisfactory stability, the available evidence has not established the consistent superiority of one construct over another. Differences in implant-related symptoms, residual laxity, graft integrity, and patient-reported outcomes therefore remain uncertain [7,8]. Direct clinical comparisons between these specific tibial fixation constructs are limited.

Because the tibial fixation strategy in our practice is strictly dictated by the initial graft diameter, the cortical button and U-staple groups inherently differ in their baseline graft thickness. Comparing patient-reported outcome measures (PROMs) and objective stability in this context is essential to determine whether adjusting the graft configuration to achieve a larger diameter with suspensory fixation yields clinical benefits that offset or surpass those of conventional full-tunnel fixation of a thinner graft. Accordingly, this study compared the clinical and magnetic resonance imaging (MRI) outcomes of quadrupled hamstring grafts secured with a tibial cortical button and doubled hamstring grafts fixed with a bioabsorbable interference screw and U-shaped staple. We hypothesized that cortical button fixation would provide objective stability and graft integrity comparable to the conventional screw-and-staple construct.

## 2. Materials and Methods

### 2.1. Study Design and Participants

This retrospective two-center comparative cohort study was conducted in the Departments of Orthopaedics and Traumatology at Sultanbeyli State Hospital and Marmara University Pendik Training and Research Hospital. The study protocol was approved by the Marmara University Faculty of Medicine Clinical Research Ethics Committee on 27 March 2026 (protocol No. 09.2026.26-0287). The study was conducted in accordance with the principles of the Declaration of Helsinki, and all patient data were anonymized before analysis.

The clinical and radiological records of patients who underwent primary ACLR between October 2021 and June 2025 were retrospectively reviewed. The procedures were performed by two orthopaedic surgeons. Surgeon A performed all procedures at Sultanbeyli State Hospital, and Surgeon B performed all procedures at Marmara University Pendik Training and Research Hospital. The tibial fixation construct was selected intraoperatively according to the diameter of the initially prepared graft. Patients were classified according to the fixation construct documented in the operative report as either the cortical button group or the bioabsorbable interference screw plus U-staple group.

Patients were eligible if they were 18–55 years of age, underwent primary ACLR using a hamstring autograft, received one of the specified tibial fixation constructs, and had a minimum postoperative follow-up of 12 months. Postoperative clinical examination findings and MRI data sufficient for outcome assessment were also required.

Patients were excluded if they had undergone previous surgery on the ipsilateral knee, required revision ACLR, or had an associated ligamentous or meniscal injury. Only patients with isolated ACL tears were included. Patients who underwent concomitant procedures, including meniscal repair or meniscectomy, osteotomy, microfracture, or meniscal root repair, were also excluded. Cases with incomplete clinical or radiological records were not included in the analysis.

After application of the eligibility criteria, 58 patients were included in the final analysis. Of these, 26 were classified into the cortical button group and 32 into the U-staple group.

### 2.2. Surgical Technique and Study Groups

All procedures were performed arthroscopically using hamstring tendon autografts. Both the semitendinosus and gracilis tendons were harvested and used in all patients. All grafts were harvested using an identical technique and standard open-ended tendon strippers at both centers. After harvesting, residual muscle tissue was removed, and the tendons were prepared together on a graft table. The combined semitendinosus–gracilis graft was initially prepared in a longer doubled configuration, and its diameter was measured using a standard sizing block. When the diameter of the initially prepared graft was 7 mm or less, the graft was reconfigured as a shorter quadrupled construct to obtain a larger final graft diameter and was used with tibial cortical button fixation. When the initial graft diameter was greater than 7 mm, the longer doubled configuration was retained and used with full-tunnel tibial fixation. The final graft diameter was measured in millimeters before implantation and recorded in the operative report. Postoperative rehabilitation was standardized across both centers. Patients were allowed immediate weight-bearing as tolerated with a hinged knee brace locked in extension. Progressive ROM exercises were initiated on postoperative day 1. Closed kinetic chain exercises began at 2 weeks, light running at 3 months, and return to sports was permitted after 9–12 months upon passing functional criteria. Routine clinical follow-ups were scheduled at 2 weeks, 6 weeks, 3 months, 6 months, 12 months, and annually thereafter.

**Figure 1:**
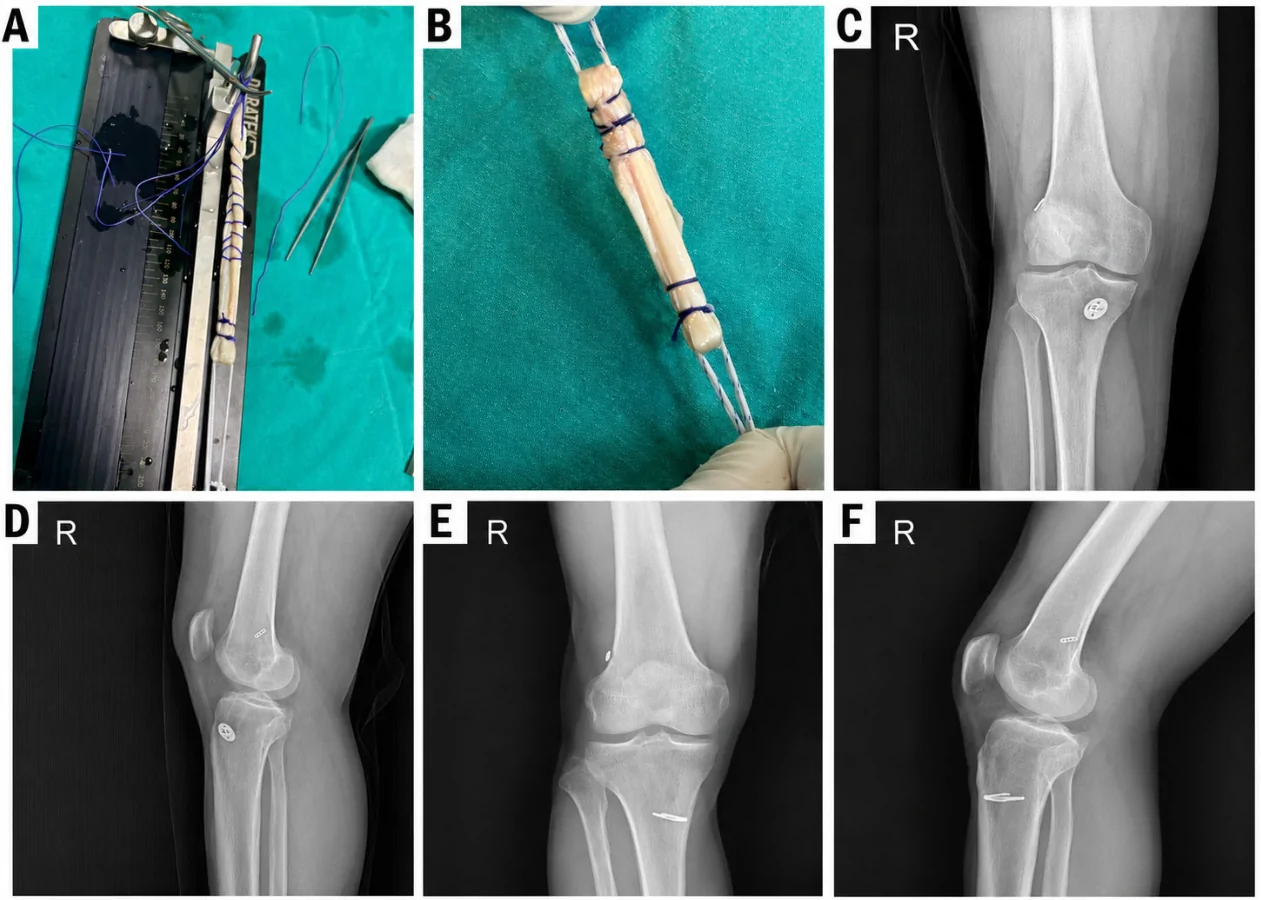
**Representative graft configurations and postoperative radiographs of the two tibial fixation constructs.** (**A**) Longer doubled hamstring graft configuration used in the U-staple group. (**B**) Shorter quadrupled hamstring graft configuration used in the cortical button group. (**C**,**D**) Anteroposterior and lateral radiographs demonstrating tibial cortical button fixation. (**E**,**F**) Anteroposterior and lateral radiographs demonstrating the U-shaped staple used to reinforce tibial fixation with a bioabsorbable interference screw. R, right side.

The general principles of femoral fixation were similar between the groups, whereas the tibial fixation strategies differed. In the cortical button group, the shorter quadrupled hamstring graft was secured to the tibial cortex using a cortical button without additional interference screw fixation. In the U-staple group, the longer doubled hamstring graft was fixed within the tibial tunnel using a bioabsorbable interference screw and reinforced with a U-shaped staple. Representative graft configurations and postoperative radiographs of the two tibial fixation constructs are shown in Figure 1.

Although the general principles of graft passage, tensioning, femoral fixation, and postoperative rehabilitation were similar, the procedures were performed at two participating centers by two orthopaedic surgeons. Therefore, center and surgeon related differences in surgical technique and perioperative management could not be excluded.

### 2.3. Clinical Assessment

Clinical outcomes were obtained from the latest available postoperative assessment performed at least 12 months after surgery. Functional outcomes were evaluated using the International Knee Documentation Committee (IKDC) subjective knee score and the Lysholm score, with higher scores indicating better knee function. Donor-site pain was assessed using the visual analog scale (VAS), which ranges from 0 to 10, specifically focusing on pain localized to the anteromedial tibial incision and harvest tract, and distinct from generalized intra-articular knee pain. Knee range of motion (ROM) was recorded in degrees as the total arc between maximal extension and maximal flexion.

Postoperative knee stability was assessed using the Lachman and pivot-shift tests. Pivot-shift findings were categorized as negative or positive. Lachman findings were recorded as negative, grade 1, grade 2, or grade 3 according to the estimated anterior tibial translation. Negative and grade 1 findings, corresponding to translation of no more than 5 mm, were considered clinically acceptable, whereas grade 2 and grade 3 findings, corresponding to translation greater than 5 mm, were classified as clinically relevant residual laxity.

### 2.4. MRI Assessment and Definition of Graft Failure

Follow-up MRI examinations were reviewed to assess graft continuity and integrity. Graft failure was defined as MRI evidence of graft discontinuity or insufficiency accompanied by clinically relevant instability, defined as grade 2–3 Lachman laxity or a positive pivot-shift test. Isolated MRI signal changes without corresponding clinical instability were not classified as graft failure.

### 2.5. Outcome Measures

Quantitative outcomes included graft-harvest site VAS score, graft diameter, IKDC score, Lysholm score, and knee ROM. Categorical outcomes comprised pivot-shift findings, Lachman classification, and graft failure determined using the predefined combined clinical and MRI criteria. The primary comparison was between two tibial fixation constructs: a shorter quadrupled hamstring graft secured with a tibial cortical button and a longer doubled hamstring graft fixed with a bioabsorbable interference screw supplemented by a U-shaped staple.

### 2.6. Statistical Analysis

Statistical analyses were performed using IBM SPSS Statistics, version 31 (IBM Corp., Armonk, NY, USA). The distribution of numerical variables was assessed before comparative analysis. As these variables did not meet the assumption of normality, they were summarized as the median and interquartile range and compared between the groups using the Mann–Whitney U test. Categorical variables, including pivot-shift findings, Lachman categories, and graft failure, were presented as *n* (%). Between-group comparisons were performed using the Pearson chi-square test, while Fisher’s exact test was applied when expected cell frequencies were less than five. All tests were two-sided, and *p* < 0.05 was considered statistically significant. Baseline demographic and follow-up variables were analyzed using the same statistical approach, with the Mann–Whitney U test used for continuous variables and the Pearson chi-square or Fisher’s exact test used for categorical variables, as appropriate.

## 3. Results

After application of the eligibility criteria, 58 patients were included in the final analysis. The cortical button group included 26 patients, and the U-staple group included 32 patients. Clinical, functional, stability-related, and graft-related outcomes were subsequently compared between the two groups.

### 3.1. Baseline Characteristics

Baseline demographic and follow-up characteristics are summarized in Table 1. The median age was 26.0 years (IQR, 21.25–31.75) in the cortical button group and 23.5 years (IQR, 22.0–27.0) in the U-staple group, with no significant between-group difference (*p* = 0.525). The groups were also comparable with respect to the operated side (*p* = 0.771). However, the proportion of male patients was significantly higher in the U-staple group than in the cortical button group (96.9% vs. 76.9%, *p* = 0.038). Follow-up duration was significantly longer in the U-staple group, with a median follow-up of 22.0 months (IQR, 19.0–26.0) compared with 17.0 months (IQR, 13.0–19.0) in the cortical button group (*p* < 0.001). The overall mean follow-up duration for the entire cohort was 19.9 ± 5.1 months.

### 3.2. Clinical and Functional Outcomes

Donor-site pain was significantly greater in the cortical button group than in the U-staple group, with median VAS scores of 2.5 (2.0–4.0) and 2.0 (0–2.0), respectively (U = 248.0, Z = −2.700, *p* = 0.007). Graft diameter was also significantly larger in the cortical button group [10.0 mm (10.0–10.0) vs. 9.0 mm (8.0–9.0); U = 156.0, Z = −4.247, *p* < 0.001]. No early postoperative complications, such as infection or deep vein thrombosis, were observed in either group.

In contrast, IKDC scores were significantly higher in the U-staple group. The median IKDC score was 60.0 (55.0–62.8) in the cortical button group and 71.0 (51.5–78.0) in the U-staple group (U = 568.5, Z = 2.387, *p* = 0.017). No significant between-group difference was observed in Lysholm scores [74.0 (68.3–79.0) vs. 76.0 (67.5–82.0); U = 440.5, Z = 0.384, *p* = 0.701] or knee ROM [160° (150–160°) vs. 160° (160–160°); U = 448.5, Z = 0.662, *p* = 0.508].

**Figure 2:**
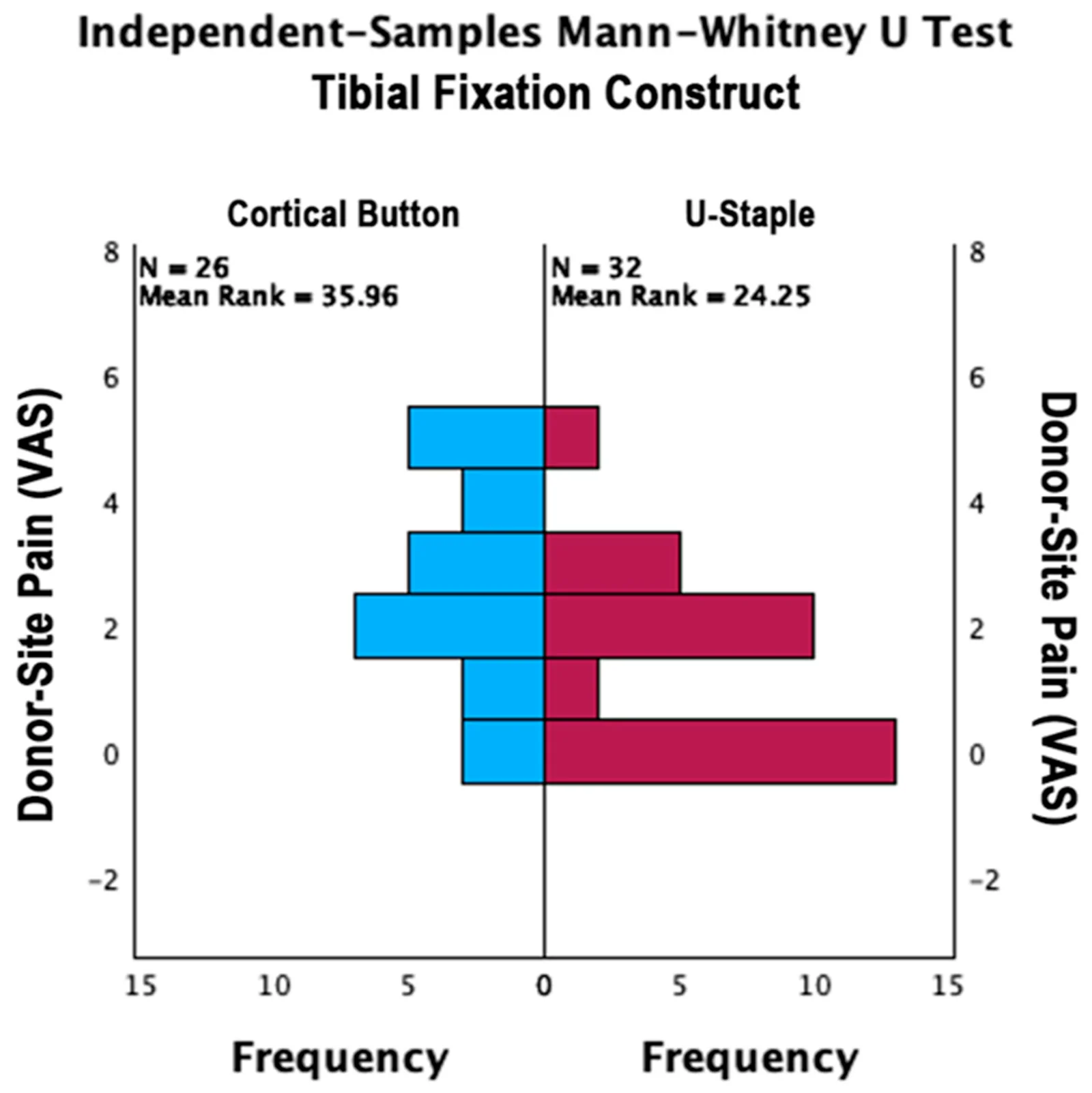
**Distribution of donor-site VAS scores according to the tibial fixation construct.** The blue bars represent the cortical button group, and the red bars represent the U-staple group. Donor-site pain scores were distributed at higher values in the cortical button group than in the U-staple group. The difference between the groups was statistically significant (Mann–Whitney U = 248.0, Z = −2.700, *p* = 0.007). VAS, visual analog scale.

**Figure 3:**
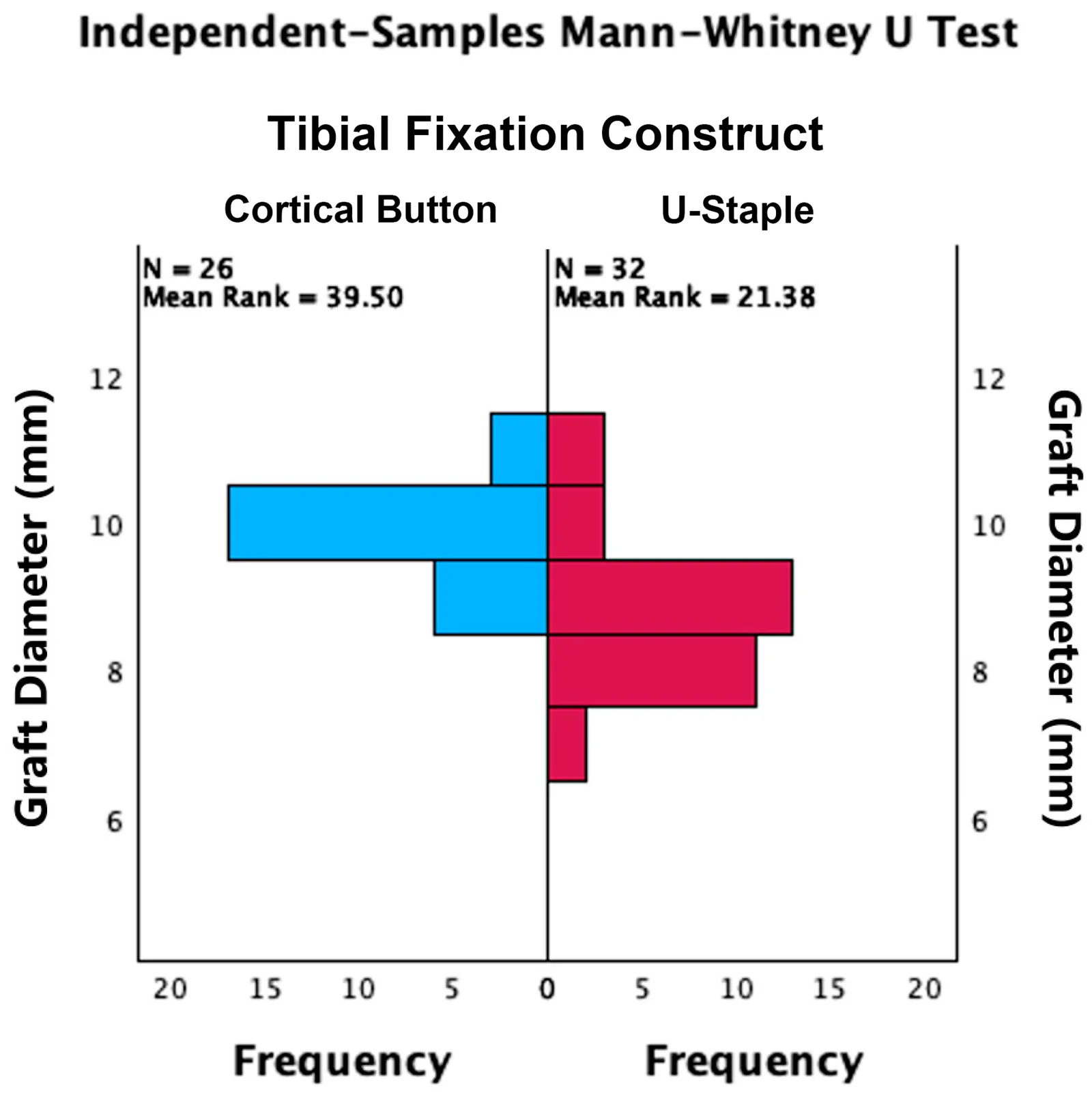
**Distribution of graft diameters according to the tibial fixation construct.** Blue bars indicate the cortical button group, whereas red bars indicate the U-staple group. Graft diameter was significantly greater in the cortical button group (Mann–Whitney U = 156.0, Z = −4.247, *p* < 0.001). Values are expressed in millimeters.

**Figure 4:**
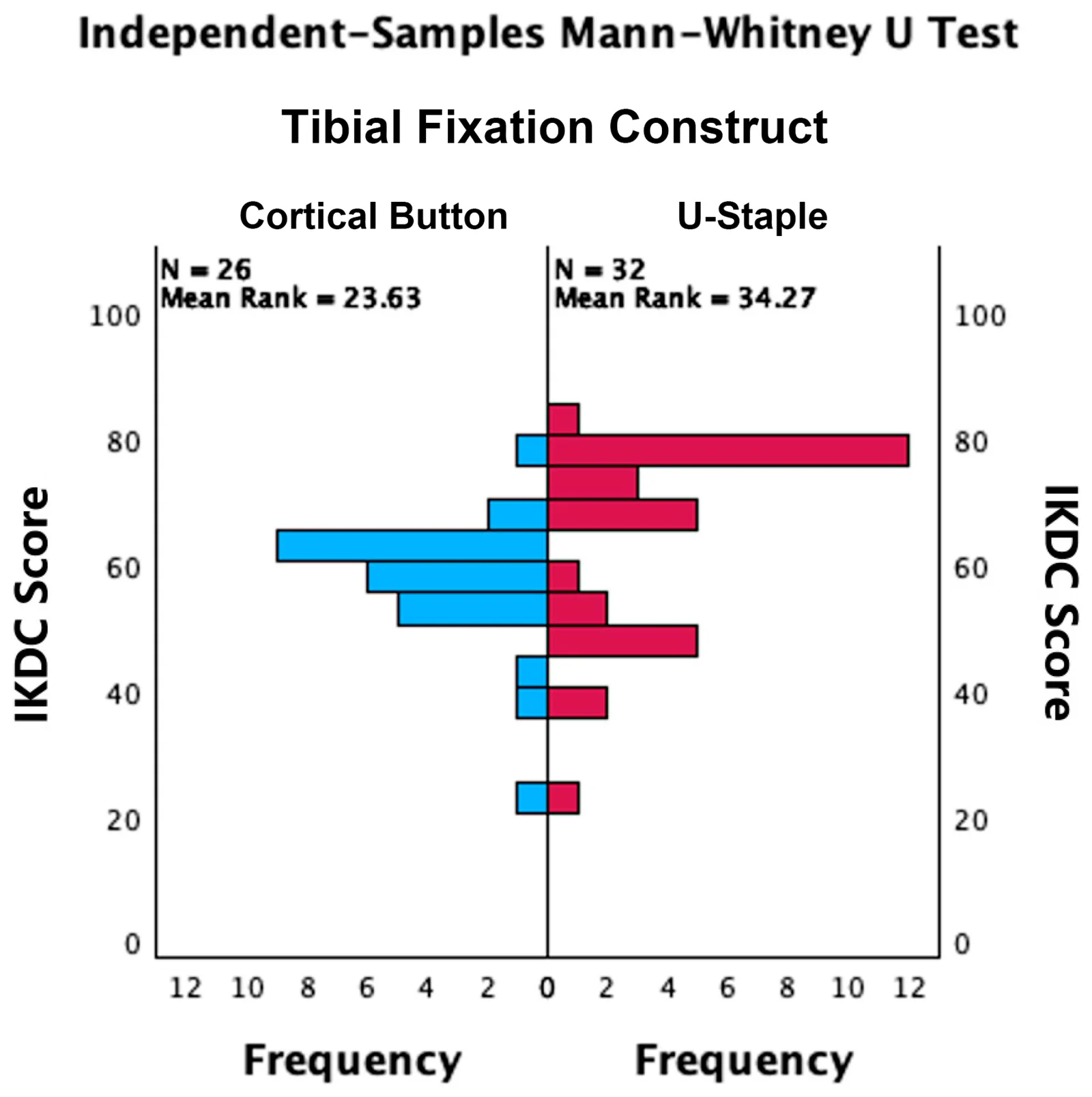
**Distribution of IKDC scores according to the tibial fixation construct.** Blue bars indicate the cortical button group, whereas red bars indicate the U-staple group. The U-staple group had significantly higher IKDC scores than the cortical button group (Mann–Whitney U = 568.5, Z = 2.387, *p* = 0.017).

The numerical outcomes are summarized in Table 2. The graphical distributions were consistent with the statistical findings: donor-site VAS scores and graft diameters tended to be higher in the cortical button group, whereas IKDC scores tended to be higher in the U-staple group, as shown in Figure 2, Figure 3 and Figure 4.

### 3.3. Postoperative Stability and Graft Failure

A positive pivot-shift test was observed in 3 of 26 patients (11.5%) in the cortical button group and in 2 of 32 patients (6.3%) in the U-staple group, with no significant between-group difference (Fisher’s exact test, *p* = 0.648).

Clinically acceptable Lachman findings, defined as a negative or grade 1 result, were present in 18 patients (69.2%) in the cortical button group and 26 patients (81.3%) in the U-staple group. Clinically relevant residual laxity, defined as a grade 2 or grade 3 result, was identified in 8 patients (30.8%) and 6 patients (18.8%), respectively. The distribution of Lachman findings did not differ significantly between the groups (Pearson chi-square test, *p* = 0.287).

Graft failure, determined using the predefined combined clinical and MRI criteria, was identified in 3 patients (11.5%) in the cortical button group and 6 patients (18.8%) in the U-staple group. This difference was not statistically significant (Fisher’s exact test, *p* = 0.495). The categorical outcomes are summarized in Table 3.

## 4. Discussion

The present study found no significant between-group differences in postoperative ROM, Lysholm scores, clinical stability, or graft failure rates after hamstring autograft ACLR. However, donor-site pain was lower and IKDC scores were higher in the U-staple group, whereas graft diameter was significantly greater in the cortical button group. Taken together, these findings do not indicate a consistent overall clinical advantage for either tibial fixation construct. The study hypothesis was therefore only partially supported, as cortical button fixation achieved comparable objective stability and graft integrity but did not provide superior patient-reported outcomes. Both the semitendinosus and gracilis tendons were routinely harvested in all patients to ensure sufficient graft volume and length, as harvesting only the semitendinosus could yield inadequate dimensions in our specific patient population.

A key methodological difference between the groups was the graft configuration. Both the semitendinosus and gracilis tendons were used in each group. In the U-staple group, the combined hamstring graft was prepared as a longer doubled construct suitable for full-tunnel fixation, whereas in the cortical button group, it was prepared as a shorter quadrupled construct compatible with suspensory cortical fixation. The quadrupled configuration allowed a greater graft diameter to be obtained despite the shorter graft length. Previous studies have shown that differences in hamstring graft configuration may affect graft dimensions without necessarily producing corresponding differences in postoperative ROM, muscle strength, knee laxity, or functional outcomes [9]. Similar principles of shorter quadrupled graft preparation have also been described previously [10]. Accordingly, the larger final graft diameter observed in the cortical button group was directly related to the intraoperative decision to convert grafts with an initially measured diameter of 7 mm or less into a shorter quadrupled configuration. Because the fixation construct was selected according to the initial graft diameter, graft configuration and fixation strategy were inherently linked, and their independent effects could not be isolated.

The two constructs were based on different fixation principles. The cortical button group used suspensory fixation of a shorter quadrupled graft on the tibial cortex, whereas the U-staple group used full-tunnel aperture fixation with a bioabsorbable interference screw reinforced by a staple. Biomechanical studies have demonstrated that cortical suspensory fixation can provide adequate resistance to cyclic loading and satisfactory ultimate failure strength, although construct stiffness and graft displacement vary according to the device and tunnel configuration [11]. Registry studies have also suggested that tibial fixation strategy may influence revision risk; however, reported associations have differed across fixation methods and patient populations [12,13]. Clinical studies evaluating screw- and staple-based constructs have generally reported satisfactory stability and functional outcomes without establishing the consistent superiority of a single fixation technique [14,15]. The absence of significant between-group differences in Lachman, pivot-shift, and graft failure outcomes similarly suggests that both constructs can provide acceptable postoperative stability.

Graft diameter has been widely investigated as a potential determinant of failure after hamstring autograft ACLR. Previous studies have associated smaller graft diameters with a higher risk of revision, particularly in younger and more active patients [16,17]. Large registry-based analyses have also identified graft size as one of several factors related to revision risk [18]. However, current evidence does not support a single universal diameter threshold as the clinical effect of graft size may vary according to patient age, sex, body size, activity level, and surgical technique [19]. In the present cohort, the median graft diameter was 10 mm in the cortical button group and 9 mm in the U-staple group, with both values above the ranges most commonly associated with increased failure risk. Despite the larger graft diameter in the cortical button group, no significant reduction in graft failure was observed, suggesting that graft diameter alone did not determine graft survival in this cohort.

Donor-site pain was significantly higher in the cortical button group; however, this finding should be interpreted cautiously. Since both tendons were harvested in all patients, this difference is unlikely related to the harvest itself. The higher donor-site pain in the cortical button group may instead be related to subtle differences in surgical technique, such as the extent of distal soft-tissue dissection required, the tensioning of the suspensory sutures, or the specific positioning of the tibial tunnel. Both the semitendinosus and gracilis tendons were harvested in each group, making gracilis preservation an unlikely explanation for the observed difference. Donor-site symptoms may instead be influenced by several factors, including the harvesting technique, incision characteristics, soft-tissue dissection, local sensory disturbance, rehabilitation, activity level, and the interval between surgery and final assessment. Because these variables were not standardized or included in the analysis, the higher VAS scores cannot be attributed directly to either the cortical button construct or the quadrupled graft configuration. In addition, donor-site pain was only assessed at the final follow-up, and the absence of serial postoperative measurements prevented evaluation of changes over time.

The U-staple group demonstrated significantly higher final IKDC scores, whereas Lysholm score and knee ROM did not differ significantly between the groups. This divergence may reflect differences in the content and responsiveness of the outcome measures. Patient-reported outcome measures may vary in their responsiveness after ACLR [20], and the IKDC and Lysholm scores are not interchangeable or equally sensitive to postoperative changes [21]. Therefore, the isolated difference in IKDC scores, without corresponding differences in Lysholm score, ROM, clinical stability, or graft failure, does not establish the overall superiority of the U-staple construct. Furthermore, because preoperative scores were unavailable, this finding represents an association at final follow-up rather than evidence of greater postoperative improvement attributable to the fixation method.

No significant between-group differences were found in clinically relevant Lachman laxity or pivot-shift findings. Although grade 2–3 Lachman laxity and positive pivot-shift results were more frequent in the cortical button group, the number of events was limited. Residual anterior or rotational laxity may adversely affect long-term patient-reported outcomes [22]; however, mild laxity does not necessarily indicate graft failure. Clinical examination findings should therefore be interpreted together with symptoms, functional instability, and imaging findings.

In the present study, graft failure was defined as MRI evidence of graft discontinuity or insufficiency accompanied by clinically relevant instability, represented by grade 2–3 Lachman laxity or a positive pivot-shift test. This combined approach was used because postoperative MRI signal abnormalities may persist despite satisfactory clinical stability and function [23]. Moreover, definitions of ACLR failure vary across the literature and may include revision surgery, graft discontinuity, recurrent instability, pathological laxity, or unsatisfactory functional outcomes [24]. Although the combined clinical and MRI criteria used in this study reduced the likelihood of classifying isolated imaging abnormalities as failure, they do not represent a previously validated composite definition. Accordingly, the reported failure rates should be interpreted within the predefined study criteria.

Graft failure was identified in 11.5% of the cortical button group and 18.8% of the U-staple group; however, this difference was not statistically significant. The numerical direction of this finding differed from that of the IKDC results, further indicating that neither construct demonstrated a consistent advantage across all outcomes. Failure after ACLR is multifactorial and may be influenced by patient age, activity level, graft dimensions, baseline laxity, posterior tibial slope, tunnel position, rehabilitation, return-to-sport exposure, and subsequent trauma [25]. Therefore, the tibial fixation construct should be considered one component of the overall reconstruction rather than an isolated determinant of graft survival.

From a clinical perspective, both tibial cortical button fixation and bioabsorbable interference screw fixation reinforced with a U-staple provided acceptable postoperative stability. The choice of construct may therefore depend on tendon length, intended graft configuration, tunnel characteristics, tibial bone quality, implant profile, cost, and surgeon preference. Although the U-staple group demonstrated higher IKDC scores and lower donor-site pain, these findings were not accompanied by significant differences in Lysholm score, ROM, objective stability, or graft failure. Likewise, the larger graft diameter observed in the cortical button group was more likely related to the shorter quadrupled graft configuration than to an independent effect of the fixation device.

These findings should be interpreted in light of several limitations. The retrospective, nonrandomized design introduced the potential for selection bias and residual confounding. Because the procedures were performed at two centers by two surgeons and the fixation construct was selected according to the initial graft diameter, the independent effects of fixation method, graft configuration, surgeon, and treatment center could not be separated. The small sample size leaves the study underpowered to detect subtle differences in low-frequency binary outcomes, such as graft failure or pivot-shift positivity, potentially introducing Type II errors. Furthermore, because the assignment of surgeons was specific to each center, this introduces a potential center/surgeon-specific bias. The baseline sex distribution and follow-up duration also differed significantly between the groups. The higher proportion of male patients and the longer follow-up duration in the U-staple group therefore represent additional potential sources of residual confounding. BMI and the exact interval from injury to surgery were not consistently available in the retrospective records and could not be included as baseline covariates. Postoperative tunnel widening was not evaluated, which could have provided additional information regarding the biomechanical behavior of the implants. Preoperative IKDC and Lysholm scores and serial postoperative donor-site VAS measurements were unavailable, and potentially relevant patient-, rehabilitation-, and follow-up-related variables could not be adjusted for. Finally, stability was assessed using manual tests without instrumented measurements, MRI assessment was not standardized or blinded, and the combined clinical and MRI definition of graft failure has not been formally validated. The exact time to graft failure could not be consistently determined from the retrospective records, precluding a time-to-failure analysis.

## 5. Conclusions

Lysholm scores, knee ROMs, postoperative stability, and graft failure rates did not differ significantly between the two tibial fixation constructs after hamstring autograft ACLR. The U-staple group demonstrated lower donor-site pain and higher IKDC scores, whereas the cortical button group had larger graft diameters. This difference in graft diameter was most likely related to the shorter quadrupled graft configuration used with cortical button fixation rather than to the fixation device itself. Overall, neither tibial fixation construct demonstrated consistent clinical superiority. Prospective randomized studies with standardized graft preparation, comparable follow-up periods, baseline functional assessments, instrumented laxity measurements, and blinded MRI evaluation are required to clarify the independent effect of each fixation strategy.

## Figures and Tables

**Table 1:** Baseline demographic and follow-up characteristics of the study groups.

Variable	Cortical Button Group (*n* = 26)	U-Staple Group (*n* = 32)	*p* Value
Age, years	26.0 (21.25–31.75)	23.5 (22.0–27.0)	0.525 *
Sex, *n* (%)			**0.038 †**
Male	20 (76.9%)	31 (96.9%)	
Female	6 (23.1%)	1 (3.1%)	
Operated side, *n* (%)			0.771 ‡
Right	12 (46.2%)	16 (50.0%)	
Left	14 (53.8%)	16 (50.0%)	
Follow-up duration, months	17.0 (13.0–19.0)	22.0 (19.0–26.0)	<0.001 *

Values for continuous variables are presented as median (25th–75th percentile), and categorical variables are presented as *n* (%). * Mann–Whitney U test. † Fisher’s exact test. ‡ Pearson chi-square test. Bold *p* values indicate statistical significance.

**Table 2:** Comparison of numerical outcomes between the groups.

Variable	Cortical Button Group (*n* = 26)	U-Staple Group (*n* = 32)	U	Z	*p* Value
Donor-site pain, VAS	2.5 (2.0–4.0)	2.0 (0–2.0)	248.0	−2.700	**0.007**
Graft diameter, mm	10.0 (10.0–10.0)	9.0 (8.0–9.0)	156.0	−4.247	**<0.001**
IKDC score	60.0 (55.0–62.8)	71.0 (51.5–78.0)	568.5	2.387	**0.017**
Lysholm score	74.0 (68.3–79.0)	76.0 (67.5–82.0)	440.5	0.384	0.701
Knee ROM, degrees	160 (150–160)	160 (160–160)	448.5	0.662	0.508

Values are presented as median (25th–75th percentile). U represents the Mann–Whitney U test statistic, and Z represents the standardized test statistic. Bold *p* values indicate statistical significance. VAS, visual analog scale; IKDC, International Knee Documentation Committee; ROM, range of motion.

**Table 3:** Comparison of postoperative stability and graft failure.

Outcome	Cortical Button Group (*n* = 26)	U-Staple Group (*n* = 32)	*p* Value
Positive pivot-shift test	3 (11.5%)	2 (6.3%)	0.648 *
Grade 2–3 residual Lachman laxity	8 (30.8%)	6 (18.8%)	0.287 **
Graft failure	3 (11.5%)	6 (18.8%)	0.495 *

* Fisher’s exact test. ** Pearson chi-square test.

## Data Availability

The data are available from the corresponding author upon reasonable request. The data are not publicly available due to patient privacy and ethical restrictions.

## References

[1] Brand J., Weiler A., Caborn D.N., Brown C.H., Johnson D.L. (2000). Graft fixation in cruciate ligament reconstruction. Am. J. Sports Med..

[2] Kousa P., Järvinen T.L., Vihavainen M., Kannus P., Järvinen M. (2003). The fixation strength of six hamstring tendon graft fixation devices in anterior cruciate ligament reconstruction. Part II: Tibial site. Am. J. Sports Med..

[3] Hill P.F., Russell V.J., Salmon L.J., Pinczewski L.A. (2005). The influence of supplementary tibial fixation on laxity measurements after anterior cruciate ligament reconstruction with hamstring tendons in female patients. Am. J. Sports Med..

[4] Teo W.W., Yeoh C.S., Wee T.H. (2017). Tibial fixation in anterior cruciate ligament reconstruction. J. Orthop. Surg..

[5] Yıldırım C., Demirel M., Koraman E., Muratoğlu O.G., Yamak F., Bozdağ S.E., Kocabey Y. (2024). The Effect of Supplementary Staple Fixation on Biomechanical Properties of Soft Tissue Graft Tibial Fixation in Anterior Cruciate Ligament Reconstruction. J. Knee Surg..

[6] Zheng F., Gao J., Wang C., Zheng X., Tang J., Zhang J., Zuo J. (2025). Impact of tibial supplementary fixation in anterior cruciate ligament reconstruction for soft tissue auto and allografts: Modest enhancement in stability and increased incidence of pain: A systematic review and meta-analysis. J. Exp. Orthop..

[7] Fu C.W., Chen W.C., Lu Y.C. (2020). Is all-inside with suspensory cortical button fixation a superior technique for anterior cruciate ligament reconstruction surgery? A systematic review and meta-analysis. BMC Musculoskelet. Disord..

[8] Lai P.J., Wong C.C., Chang W.P., Liaw C.K., Chen C.H., Weng P.W. (2022). Comparison of two different types of hybrid Tibial fixations for anterior cruciate ligament reconstruction: A prospective comparative cohort study. BMC Musculoskelet. Disord..

[9] Mo I.F., Harlem T., Faleide A.G.H., Strand T., Vindfeld S., Solheim E., Inderhaug E. (2024). ACL Reconstruction Using Quadrupled Semitendinosus Versus Double-Stranded Semitendinosus and Gracilis Autograft: 2-Year Results From a Prospective Randomized Controlled Study. Am. J. Sports Med..

[10] Martinez-Cano J.P., Zamudio-Castilla L.M., Cuadros-Potes J.A., Ibarra-Mosquera L.C., Mejia-Lopez F.M. (2019). Quadrupled Semitendinosus ACL Reconstruction Combining Cortical Button in Femur and Interference Screw in Tibia. Arthrosc. Tech.

[11] Ammann E., Hecker A., Bachmann E., Snedeker J.G., Fucentese S.F. (2022). Evaluation of Tibial Fixation Devices for Quadrupled Hamstring ACL Reconstruction. Orthop. J. Sports Med..

[12] Rahardja R., Love H., Clatworthy M.G., Monk A.P., Young S.W. (2022). Suspensory Versus Interference Tibial Fixation of Hamstring Tendon Autografts in Anterior Cruciate Ligament Reconstruction: Results From the New Zealand ACL Registry. Am. J. Sports Med..

[13] Persson A., Gifstad T., Lind M., Engebretsen L., Fjeldsgaard K., Drogset J.O., Forssblad M., Espehaug B., Kjellsen A.B., Fevang J.M. (2018). Graft fixation influences revision risk after ACL reconstruction with hamstring tendon autografts. Acta Orthop..

[14] Carulli C., Matassi F., Soderi S., Sirleo L., Munz G., Innocenti M. (2017). Resorbable screw and sheath versus resorbable interference screw and staples for ACL reconstruction: A comparison of two tibial fixation methods. Knee Surg. Sports Traumatol. Arthrosc..

[15] De Wall M., Scholes C.J., Patel S., Coolican M.R., Parker D.A. (2011). Tibial fixation in anterior cruciate ligament reconstruction: A prospective randomized study comparing metal interference screw and staples with a centrally placed polyethylene screw and sheath. Am. J. Sports Med..

[16] Magnussen R.A., Lawrence J.T., West R.L., Toth A.P., Taylor D.C., Garrett W.E. (2012). Graft size and patient age are predictors of early revision after anterior cruciate ligament reconstruction with hamstring autograft. Arthroscopy.

[17] Spragg L., Chen J., Mirzayan R., Love R., Maletis G. (2016). The Effect of Autologous Hamstring Graft Diameter on the Likelihood for Revision of Anterior Cruciate Ligament Reconstruction. Am. J. Sports Med..

[18] Snaebjörnsson T., Hamrin-Senorski E., Svantesson E., Karlsson L., Engebretsen L., Karlsson J., Samuelsson K. (2019). Graft Diameter and Graft Type as Predictors of Anterior Cruciate Ligament Revision: A Cohort Study Including 18,425 Patients from the Swedish and Norwegian National Knee Ligament Registries. J. Bone Jt. Surg. Am..

[19] Mirzayan R., Chang R.N., Royse K.E., Reyes C.E., Prentice H.A., Maletis G.B. (2025). Is There a Hamstring Autograft Diameter Threshold for Anterior Cruciate Ligament Reconstruction?. Orthop. J. Sports Med..

[20] Abed V., Kapp S., Nichols M., Castle J.P., Landy D.C., Conley C., Stone A.V. (2024). Lysholm and KOOS QoL Demonstrate High Responsiveness in Patients Undergoing Anterior Cruciate Ligament Reconstruction: A Systematic Review and Meta-analysis of Randomized Clinical Trials. Am. J. Sports Med..

[21] Risberg M.A., Holm I., Steen H., Beynnon B.D. (1999). Sensitivity to changes over time for the IKDC form, the Lysholm score, and the Cincinnati knee score. A prospective study of 120 ACL reconstructed patients with a 2-year follow-up. Knee Surg. Sports Traumatol. Arthrosc..

[22] Sundemo D., Sernert N., Kartus J., Hamrin Senorski E., Svantesson E., Karlsson J., Samuelsson K. (2018). Increased Postoperative Manual Knee Laxity at 2 Years Results in Inferior Long-term Subjective Outcome After Anterior Cruciate Ligament Reconstruction. Am. J. Sports Med..

[23] Saupe N., White L.M., Chiavaras M.M., Essue J., Weller I., Kunz M., Hurtig M., Marks P. (2008). Anterior cruciate ligament reconstruction grafts: MR imaging features at long-term follow-up--correlation with functional and clinical evaluation. Radiology.

[24] Aldag L., Dallman J., Henkelman E., Herda A., Randall J., Tarakemeh A., Morey T., Vopat B.G. (2023). Various Definitions of Failure Are Used in Studies of Patients Who Underwent Anterior Cruciate Ligament Reconstruction. Arthrosc. Sports Med. Rehabil..

[25] Zhao D., Pan J.K., Lin F.Z., Luo M.H., Liang G.H., Zeng L.F., Huang H.T., Han Y.H., Xu N.J., Yang W.Y. (2023). Risk Factors for Revision or Rerupture After Anterior Cruciate Ligament Reconstruction: A Systematic Review and Meta-analysis. Am. J. Sports Med..

